# Connecting With Fundamental Mathematical Knowledge Directly: The Organizational Features of Good Mathematical Cognitive Structure

**DOI:** 10.3389/fpsyg.2018.02267

**Published:** 2018-12-04

**Authors:** Zezhong Yang, Yanqing Zhang, Kai Wang, Ming Zhu

**Affiliations:** The School of Mathematics and Statistics, Shandong Normal University, Jinan, China

**Keywords:** mathematical knowledge, cognitive structure, top students, connection, social network analysis

## Abstract

This paper reported on the study of a good mathematical cognitive structure (GMCS) based on 43 top university students and 82 concepts of Calculus materials, using the social network analysis method. The results indicated that the GMCS has the following organizational features: (1) The mathematical knowledge (MK) in GMCS interconnected widely, especially in MK with a higher connection tightness; (2) Most connections between MK were direct; (3) MK of the basic and higher inclusive level had a greater impact; and (4) There were multiple MK accumulation points connecting others to form subsets. These new findings enrich the results of previous GMCS studies and promotes further exploration of GMCS. In view of this, teachers should pay closer attention to basic and abstract MK and help their students construct various direct connections of the MK in their mind.

## Introduction

To describe the storage and the organization of internalized mathematical knowledge (MK) in the mind, the concept of mathematical cognitive structure (MCS) was proposed, based on the general cognitive structure concept in the field of psychology, in the 70s (Geeslin and Shavelson, 1975; Cao and Cai, 1989). It was subsequently classified into the general MCS and the good mathematical cognitive structure (GMCS) (Yang, 1993; Wu and Guo, 1997; Guan, 1998).

The so-called GMCS is the cognitive structure which contributes to an individuals’ mathematic learning and could guide individuals to understand MK correctly, quickly and to flexibly extract this knowledge to solve problems (Li, 2001; Tsai, 2001; He, 2002; Tu, 2003; Wang and Wang, 2004; Yu, 2004a; Wang and Zheng, 2008; Yu et al., 2011; Alison et al., 2013; Wang, 2013). Consequently, GMCS has been a hot topic in the field of mathematics education and has attracted much attention for a long time (Yang, 1993; Zhang and Wu, 2012). A series of studies on GMCS, especially on its characteristics, have been conducted over the past 30 years (Sun and Yang, 2015).

These studies were based on an extensive and firm belief in the field of mathematic education, that the MCS was developed during learning activities and when applying MK. Once the characteristics of GMCS were discovered, teachers could provide purposeful guidance to form aGMCS in mathematical teaching, and could rapidly enhance the quality of teaching (Bruner, 1989; Ausubel, 1994; Dixon, 2005; Zhang, 2007; Zhang and Wu, 2012; Sofia et al., 2017).

However, reviewing these studies, it was found that the majority of studies conducted on the basis of a simple investigation or theoretical speculation and many results put forward had obvious subjectivity and inaccuracy and were generally difficult to impossible to achieve in mathematical teaching. Furthermore, some aspects or questions on GMCS have not been addressed, such as questions on how mathematical theorems, problems, and graphics are organized in GMCS. It is necessary to further explore the GMCS based on previous studies.

### Literature Review

Since the 1970s, the study of GMCS have continued. In reviewing these studies, it was found that they mainly focus on knowledge and its organization in the GMCS.

#### The Knowledge in the GMCS

Previous studies have primarily focused on knowledge in a GMCS (Tsai, 2001). However, currently most researchers believe that GMCS should include a rich MK, that is to say, it should be a vast storehouse of knowledge. In particular, it should include more knowledge on production and problem-solving strategies (Guan, 1998; He, 2002; Qu and Yang, 2018). Productive knowledge refers to a knowledge series composed of conditions and related actions. It is an action which occurs when certain conditions are met (He, 2002; Kong and Zeng, 2009). The problem-solving strategy refers to the knowledge that a person understands the problem condition, chooses the method of solving the problem and determines the procedure of solving the problem, in the process of solving the problem (Li, 1998). Some researchers believe that GMCS should also include knowledge of a relational representation system and a conceptual representation system. The relational representation system is the individual’s understanding of the relationship to MK. The concept representation system refers to the understanding of the relationship between the individual and the knowledge (Wang and Zheng, 2008). Moreover, some researchers also believed that a GMCS should include more meta-cognitive knowledge (Mi and Hou, 2011).

For MK, most researchers agree that it should be clear, stable, flexible, individualized, and widely connected (Jin, 2002, 2011; Wang and Wang, 2004; Han and Wang, 2005; Yan and Huang, 2005; Wang and Zheng, 2008; Wang, 2013). Flexibility means that the representation of the same knowledge in a GMCS is manifold. The explanation is that MK in a GMCS should have individual characteristics. The wide connection refers to the many interconnections of MK. Some researchers also believe that MK in a GMCS should have a higher degree of “Internalization,” that is to say, the knowledge acquired after the individual’s deep processing (Yan and Huang, 2005; Mi and Hou, 2011). Some researchers believe that more connections between knowledge in a GMCS, and the different representations of the same MK should be interrelated (Wang and Wang, 2004). Moreover, some researchers believe that GMCS have the following three characteristics: (1) it can quickly absorb new knowledge; (2) it can be invoked flexibly; and (3) it can create new knowledge independently (Chen, 2003). A recent study suggested that MK is correct and rational (Qu and Yang, 2018).

#### The Organization of MK in the GMCS

Almost all researchers believe that MK in a GMCS are organized in a three-dimensional network (Papert, 1993; Guan, 1998; He, 2002; Wilkerson-Jerde and Wilensky, 2011), and have various different ideas on how this network is formed.

Some researchers believe that the network is well organized, that is to say, knowledge of the highest inclusive level is in the central position, and knowledge of a low inclusive level has a marginal position (Yan and Huang, 2005; Jin, 2011; Mi and Hou, 2011; Zhao, 2013). Knowledge of the highest inclusive level has a high degree of abstraction, which generally contains specific knowledge, for example, the concept of function. Knowledge of a low inclusive level is intuitive and the specific, such as the mathematical concept of a cube.

Some researchers believe that the organization of a GMCS is a “Standard Pyramid” in form. Abstract and generalized knowledge is at the top, while intuitive and specific knowledge is at the bottom. The higher the MK is in the pyramid, the more abstract it is. The lower the MK is in the pyramid, the more specific it is (Wang and Zheng, 2008).

Some researchers believe that the network is composed of many MK domains and systems. The MK domain is a network based on a mutual equivalence relationship. The MK system is a chain based on an abstract or reasoning relationship. This network is therefore uneven, and contains many small collections and MK clusters (Yu et al., 2011).

In reviewing previous studies on the above aspects of a GMCS, it was found that the existing achievements of a GMCS were relatively rich (Lu and Yu, 2010; Sun and Yang, 2015). However, it does not mean that it is complete, as apparent problems, such as inaccuracy and ambiguity and a disagreement on the organization of MK in a GMCS, still remain.

### Theoretical Foundation

#### The Basic Characteristics of the MCS

The MCS is an internal structure composed of a vast MK. When its contents are processed in an abstract way, that is, mathematical concepts, symbols, graphics, formulas, axioms, theorems, etc., are represented by points, and the relationship between them is represented by lines. Currently they are generally described as a big and complex networks consisting of many nodes and connections (Papert, 1993; Wilkerson-Jerde and Wilensky, 2011). This network could be classified into three basic forms. The first is the linear structure, the second is the tree structure, and the third is the three-dimensional network structure (Li, 2001; Yu, 2004b). These three basic structures could be combined with each other to form a solid integrated structure.

In MCS, the number of nodes and connections, the connection tightness and how they are connected varies from person to person. At present, researchers generally believe that the number of nodes and connections in MCS of students performing well in mathematics are more and closer together than the number of nodes and connections in MCS of other students (Wang and Wang, 2004). With regard to the distribution of nodes in the MCS, researchers currently believe that they should be uneven overall. MCS can be classified into many centralized substructures that form around one or several important concepts or theorems (Yu, 2004b; Yu et al., 2011).

Certainly, there are some researchers who believe that the nodes in MCS are stacked in layers. Among them, the nodes representing abstract and basic MK are located at the top level, and nodes representing specific MK were located at the lower level (Wo, 2000; Wang and Zheng, 2008). However, this explanation has not yet been widely accepted in academic circles (Zhang and Chen, 2000).

#### The Exploration of Cognitive Structure

For a long time, the concept map method was considered the most effective and convenient way to detect a cognitive structure (Fenker, 1975; Novak et al., 1983; Lomask et al., 1992; Ruiz-Primo and Shavelson, 1996). It can not only clearly check the amount of knowledge in the MCS, but also detect the organization of MK (Preece, 1976; Zhang and Chen, 2000; Ifenthaler et al., 2001; Tsai, 2001). However, the concept map method is not without its limitations. The lack of quantitative analysis is an obvious limitation (Zhang and Chen, 2000; Ifenthaler et al., 2001). Therefore, in recent years researchers have developed a variety of new methods to detect cognitive structures, such as the flow-map method (Anderson and Demetrius, 1993; Sofia et al., 2017). The use of these new methods not only enriched MCS study results but also greatly advanced the deep exploration of it.

Since the 1990s, the social network analysis method has been applied in the field of psychology (Xu et al., 2011). This method is based on data relation, to analyze the characteristics of a network. Its biggest advantage is that it can analyze the network relationship quantitatively and give more accurate values to the network (Liu, 2009). It was originally used for social relations analysis and mainly used to explore the density, centrality, and subgroups of social networks, so as to understand the differences between networks (Luo, 2010). This method was introduced into psychological research because there were many similarities between psychological relationship networks and social relations networks. In fact, if the specific and actual meaning represented by the elements both in the psychological network and social network was excluded, then the two types of networks were almost the same (Ma et al., 2011). Both are complex networks composed of nodes and connections. Presently, in some studies on psychological problems, the feasibility and effectiveness of social network analysis methods have been verified, and these studies have also obtained many meaningful results (Xu et al., 2011; Hou et al., 2014).

As a type of MCS, a GMCS is composed of a vast MK and can be described as a big and complex network consisting of many nodes and connections, therefore, it is feasible and appropriate to use the social network analysis method when studying GMCS (Sun, 2017). Furthermore, by introducing this new method, it is obvious that we can explore more aspects about GMCS, such as its density, centrality, and subgroups, etc., and obtain some accurate values of a GMCS to conduct a quantitative analysis, so as to solve the fuzzy problems mentioned above (Sun and Yang, 2017; Qu, 2018). Therefore, this study intends to apply the social network analysis method to analyze a GMCS, and the organizational characteristics of a GMCS. The question in this study is: What are the characteristics of MK in a GMCS through quantitative analysis? In other words: How is MK organized in a GMCS? Because a new method and a quantitative analysis is used in this study, we believe more clear results and new discoveries will be obtained, based in previous studies.

## Materials and Methods

### Participants

This study selected 43 top students mathematic students from 10 key universities in the top 20 Chinese universities as participants. Twenty-four boys and 19 girls were selected, with an average age of 19.87 years (MD = 0.13). The reason for selecting these students was that top mathematic students are generally considered to have a GMCS (Wang and Wang, 2004; Wang, 2013). The selection of top students in this study was based on the students’ usual mathematics learning characteristics and achievements. The top students were usually positive and efficient in learning, reasonable in method, excellent in performance and relatively stable (Maker, 1981; Johnson, 2000; National Council of Teachers of Mathematics, 2000). This is the popular international standard choice for top students.

The specific methods of selecting students in this study were as follows: (1) Randomly selected one class at each university and selected the top 10 students in the class according to the results of the final mathematics examination of the first semester. In this way, 100 university students were elected at first. (2) To determine the stability of their mathematics learning their ranking of mathematics scores on the last midterm exam were analyzed. Students, who did not always rank in the top 10, were then removed from the study. A total of 31 students were removed in this step. (3) Through interviews, students revealed their attitude toward mathematics and those who were not active students, were also removed. After this, 26 more students were removed.

### Instrument

This study selected 82 mathematical concepts of Calculus from the textbook of higher mathematics, published by the China Higher Education Press in 2007, as research materials. These 82 concepts are: Real number, Rational number, Density, Irrational number, Number axis, Infinite decimal, Interval, Neighborhood, Left-right neighborhood, Hollow neighborhood, Boundedness, Bounded set, Supremum (infimum), Function, Domain, Range, Mapping, Original, Composite function, Inverse function, Monotone function, Parity function, Periodic function, Basic period, Sequence of number, Subsequence, General term, Divergent, Convergence, One-sided limit, Infinitesimal, Infinity, Infinitesimal of higher order, Equivalent infinitesimal, Limit of sequence, Asymptote, Increment, Continuous, Left and right continuous, Discontinuity point, Limit of function, Uniformly continuous, Local number preserving, Derivative, One-sided derivatives, Derivative function, Derivable function, Extremum, Left (right) derivative, Extremum point, Stable point, Maximum (minimum) value, Smooth curve, Higher-order derivative, Differential, Higher-order differential, Convex (concave) function, Inflection point, Closed nested interval, Accumulation point, Open coverage, Finite coverage, Primitive function, Indefinite integral, Rational, Definite integral, Newton-Leibniz formula, Curved trapezoid, Riemann integral, Upper (lower) limit integral, Integrable function, Variable limited integral, Reductive integral, T model segmentation, Distribution integral, Riemann sum, Integral remainder term, Improper integral, Flaw point, Flaw integral, Absolutely convergent, Conditionally convergent.

The reason these concepts were chosen was because of the many connections between them. Participants had completed the learning of these concepts in the first semester of the first academic year. Therefore, these mathematical concepts helped researchers to reveal organizational characteristics of MCSs accurately and conveniently (Sofia et al., 2017).

### Data Collection

The specific process of data collection was as follows: (1) Gave students 5 min to recollect the concepts of Calculus that they learned in the first semester, and then gave students a piece of paper with the above 82 mathematical concepts to confirm them again; (2) Let the students observe these concepts, and then, according to their own understanding, link the related concepts with a line on the paper. (3) At the same time, students were required to mark the tightness of each relationship with the integers 1 to 5. The compact relationships were marked with a larger number, and the incompact relationships were marked with a smaller number.

Before drawing out the relationship between concepts, the purpose of letting students recollect the concepts and confirm all concepts, was to allow them to create a clear impression of the concepts they learned in the previous semester so that they could focus on the relationship between concepts in the following step.

In order to collect data expediently and effectively, we adopted the cluster sampling method with the student’s permission. In the survey, all freshmen from all 10 classes were invited to participate in the above activities. There were 433 students in these 10 classes, including 212 boys and 221 girls. 43 Top students, 294 middle-level students, and 96 general students. The general level students were the last 10 students in the last semester final exams. With the exception of top students and general students, the rest were regarded as the middle-level students.

This study was conducted in accordance with the recommendations of “The guidelines of the International Committee of Medical Journal Editors and the Adolescent Mental Health Specialized Committee of Chinese Mental Health Association.” Before starting the study, we obtained written informed consent from all parents of adult participants and all parents of non-adult participants. All written informed consent that the parents gave was in accordance with the Declaration of Helsinki. This study was approved by the ethics committee of Shandong Normal University School of psychology, Shandong Normal University.

### Data Analysis

This study initially collected 433 conceptual diagrams. After eliminating 27 of these incorrect and unordered concept maps (12 by middle drawing and 15 by general rendering), 406 diagrams remained and could be used for further analysis. The reason why we kept the diagrams of middle level and general students was because we intended to make a comparison between them with the diagrams of top students to find more features of a GMCS.

To facilitate the analysis with the network analysis methods, we first extracted the numbers representing the tightness of the connection from the above 406 relational diagrams, transformed it into one-mode multi-valued relations matrices, and calculated matrices of students at three different levels, respectively. Then, we analyzed the whole density, condensed subgroup, individual density and individual centrality of the MCS with the help of the network analysis software Ucinet 6.0.

The so-called whole density is the ratio of the actual relationship between members in the network to the theoretical relationship. The condensed subgroup is a subset formed by members through the interconnection. Individual density refers to the number of connections between each member and other members in the network. Individual centrality refers to the number of relationships formed by the direct connection between network members and other members (Liu, 2009; Luo, 2010). Therefore, we believe that the analysis of these four aspects would help to understand the organizational characteristics of a GMCS.

## Results

### The Whole Density

The formula for calculating the whole density of the network is 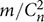 (m is the actual relation number, n is the number of members). Therefore, what the whole density revealed is the relative relation number in a network (Liu, 2009; Luo, 2010). In order to see the whole density of the GMCS clearly, we selected five tightness values 0, 1, 2, 3, and 4 as the critical value at the time of calculation, and the calculation was carried out in five times.

When the critical value was 0, all connections were considered. When the critical value was 1, only those relationships whose connection tightness was not less than 1 were considered. When the critical value was 2, only those relationships whose connection tightness was not less than 2 were considered, and so on. The results are shown in Table 1.

**Table 1:** The whole density value.

Critical value	Top students	Middle students	General students
0	0.1822	0.1647	0.1533
1	0.0410	0.0211	0.0181
2	0.0160	0.0105	0.0000
3	0.0087	0.0000	0.0000
4	0.0024	0.0000	0.0000

Table 1 shows that the whole density of the MCS of top students was higher than that of middle and general students in five cases. When the critical value was 1, the overall density of the MCS of the top students was about twice as high as that of middle and general students. When the critical value was 3 and 4, the density of the MCS of top students were 0.0087 and 0.0024, respectively, and the density of the MCS of middle and general students was 0. Therefore, the 82 concepts in the MCS of the top students had more connections than in the MCS of middle and general students, especially those concepts with a higher degree of tightness.

### The Individual Density

The individual density of members in the network refers to the number of connections formed by the member and other members of the network, so the characteristics of each individual in the network is revealed (Liu, 2009; Luo, 2010). Through the calculation, it was found that the individual density of concepts in the MCS of the top students ranges from 4 to 36, with an average of 14.7561. Of which the Function concept had the maximum individual density value. The individual density of concepts in the MCS of middle students was between 7 and 29, with an average value of 12.2415. Of which the function concept had the maximum individual density. The individual density of concepts in the MCS of general students was between 5 and 33, with an average value of 10.4146. Of which the function concept had the maximum individual density. Thus, the individual density value of the concept in the MCS of the top students was generally greater than the individual density of the concepts in the MCS of middle and general level students. Not only that, but there was actually a statistically significant difference between them (*t* = 1.955, *p* = 0.041; *t* = 2.957, *p* = 2.957). The connections between a concept and other concepts in the MCS of top students were more than the connections between a concept and the other concepts in a middle and general level students’ MCS.

In these three types of MCS in the three levels of students, the concept of function revealed the most connections with other concepts, that is to say, it was the largest accumulation point in these three types of MCS.

It should be noted that in addition to the concept of function, there were about 20 concepts in the MCS of top students, with an individual density greater than 20. These 20 concepts were: Real number, Rational number, Irrational number, Density, Infinite decimal, Interval, Neighborhood, Left-right neighborhood, Hollow neighborhood, Supremum (infimum), Primitive function, Definite integral, Newton-Leibniz formula, Curved trapezoid, Riemann integral, Upper (lower) limit integral, Integrable function, Variable limited integral and T model segmentation. In the MCS of middle and general level students, the number of concepts with an individual density greater than 20 was only 5. Therefore, the number of large accumulation points in the MCS of top students was obviously more than that of the middle and the general students.

Moreover, by analyzing these concepts of large accumulation points in MCS of top students carefully, it was found that they were all basic concepts of Calculus, and most of them had a higher inclusive level just like the concept of the Function, such as function of the Real number, Interval, Riemann Integral and Integrable. The basic concepts with a higher inclusive level therefore play an important role in MCS of the top students. There was a wide connection between these concepts and other concepts.

### The Condensed Subgroups

The condensed subgroup of the network was a subset of the interconnected members of the network. It was a maximal complete subgraph that reflected the local packet characteristics of the network (Liu, 2009; Luo, 2010). In order to get a more detailed understanding of the condensed subgroup, we also chose five tightness values 0, 1, 2, 3, and 4 as critical values and calculated the three average matrices, five times, with the Ucinet 6.0 software.

The number of condensed subgroups found in the MCS of students at different levels and the number of subgroups containing more than three concepts is shown in Table 2.

**Table 2:** The condensed subgroups.

Critical value	Subgroups	Top students	Middle students	General students
0	Numbers of subgroups	1	1	1
	Numbers of subgroups which concepts were more than three	1	1	1
	Numbers of concepts in the biggest subgroup	82	82	82
1	Numbers of subgroups	7	25	31
	Numbers of subgroups which concepts were more than three	5	14	13
	Numbers of concepts in the biggest subgroup	49	11	9
2	Numbers of subgroups	37	49	62
	Numbers of subgroups which concepts were more than three	10	10	6
	Numbers of concepts in the biggest subgroup	13	5	3
3	Numbers of subgroups	54	77	82
	Numbers of subgroups which concepts were more than three	8	1	0
	Numbers of concepts in the biggest subgroup	9	3	1
4	Numbers of subgroups	74	82	82
	Numbers of subgroups which concepts were more than three	1	0	0
	Numbers of concepts in the biggest subgroup	3	0	0

Table 2 shows that when the critical value was greater than one, the number of condensed subgroups in the MCS of the top students was always less than the number of condensed subgroups in the other two level students’ MCS.

In the MCS of top students, the number of condensed subgroups with more than three concepts was always larger than that of condensed subgroups with more than three concepts in the MCS of middle and general students.

The number of concepts in the largest condensed subgroup in the MCS of the top students was always greater than that in the largest subgroup in the MCS of the middle and general level students. When the critical value was 1, there was a condensed subgroup with a concept number of 49 in the MCS of top students, which contains the 59.8% of all concepts.

Therefore, there should be more connected concepts in the MCS of the top students than in the other two level students’ MCS. Most concepts in the MCS of top students were connected to each other, especially those with the higher tightness.

### The Individual Centrality

The individual centrality of a member in a network refers to the number of relationships formed by the member’s direct connection with other members. Therefore, what it revealed is the influence or power of members of the network (Liu, 2009; Luo, 2010).

After calculation, we found that the individual centrality of the concept in the MCS of the top students was between 3.488 and 34.474, with an average value of 11.544. The individual centrality of the concept in the MCS of the middle level students was between 1.926 and 21.545, with an average value of 6.148. The individual centrality of the concept in the MCS of the general students was between 1.171 and 14.106, with an average value of 4.544. The individual centrality of concepts in the MCS of the top students was generally greater than that of the concepts in the MCS of the middle and general level students. It could be seen that each concept has more influence in the MCS of the top students than in the MCS of the middle-level students and the general level students.

The concepts whose individual centrality were in the top 10, respectively, in the three different level students’ MCS and its average values of individual centrality were shown in Table 3.

**Table 3:** The degree of individual centrality.

Ordinal number	Top students		Middle students		General students	
	Concepts	Degree	Concepts	Degree	Concepts	Degree
1	Function	34.474	Function	21.545	Function	14.106
2	Neighborhood	24.922	Real number	12.889	Real number	9.540
3	Real number	23.979	Derivative	11.687	Convergence	7.905
4	Rational number	21.459	Sequence of number	11.081	Rational number	7.765
5	Irrational number	20.981	Definite integral	10.145	Continuous	6.999
6	Newton-Leibniz formula	19.573	Neighborhood	10.114	Neighborhood	6.917
7	Definite integral	19.531	Convergence	9.291	Irrational number	6.872
8	Interval	18.482	Limit of function	9.132	Derivative	6.833
9	Mapping	18.330	Continuous	8.981	Derivable function	6.815
10	Hollow neighborhood	18.275	Irrational number	8.970	Sequence of number	6.807

As seen in Table 3, although the MCS of students at different levels had large individual centrality of the function concept, its value was different. This reveals that the influence of the concept of function in three different MCS should be different. The influence of function concept in the MCS of top students was greater than that in other students’ MCS. In addition to the concept of function, there were four concepts in the MCS of the top students where the values of the individual centrality were more than 20. These were: Neighborhood, Real number, Rational number and Irrational number. Therefore, there are many influential concepts in the MCS of the top students.

Additionally, by analyzing these four concepts with higher influence carefully, it was found that they were all the most basic concepts of Calculus. Therefore, the basic concepts in the MCS of the top students are usually of great influence.

## Discussion

The whole density of a network is the ratio of the number of actual relationships and the number of theoretical relationships in this network. as discussed above, it can be inferred that the whole density of the MCS of top students was higher than that of middle and general students, especially when the larger degree of connection tightness value was considered. Therefore, the connection between the 82 concepts in the MCS of the top students should be more connected than the concepts in MCS of the middle and general level student, especially those connections with high tightness.

Individual density is the connections number of a member of a network connecting with other members. What it reveals is the nature of a single member in a network. From the above analysis, it can be inferred that the individual density of concepts in the MCS of the top students was generally higher than that of the concepts in the MCS of the middle and general level students. This illustrated that the number of members in the MCS of the top students was better connected than the concepts in the middle and general cognitive structures. also It was also revealed that the number of large accumulation points in the MCS of the top students was more than that in the middle and general students’ MCS, and the concepts of large accumulation points were usually basic in Calculus and had a higher degree of inclusion. This suggests that the concepts in the MCS of the top students generally connected with the many basic and high inclusive level concepts.

The network condensed subgroup is a subset formed by some interrelated concepts in the network. The above analysis revealed that there were fewer condensed subgroups in the MCS of the top students than in the middle and general level students. While the condensed subgroups contained at least three members, the MCS of top students were more than that in MCS of middle and general level students. As a result, most mathematical concepts in the MCS of the top students should be gathered together, especially those with a high degree of tightness, which should be very closely connected to each other.

The individual centrality refers to the connection number of a member of a network connecting with others directly. The above analysis revealed that the individual centrality of the concepts in the MCS of the top students, was generally greater than the individual centrality in the MCS of the middle and general level students. This showed that in the MCS of top students, the concepts directly related to other mathematical concepts were more than those in the MCS of middle and general level students. From this, we know that many mathematical concepts had a strong influence on the MCS of top students. In addition, the analysis also revealed that there were multiple influential concepts in the MCS of the top students. These concepts were also the most basic and had a higher inclusive degree in Calculus. Therefore, the basic concepts with a higher inclusive degree in the MCS of the top students, should be the most influential.

## Conclusion

Good mathematical cognitive structure plays an important role in individual mathematical activities (Skemp, 1971; Yang, 1993; Ausubel, 1994; Zhang, 2003, 2007; Dixon, 2005; Zhang and Wu, 2012; Sofia et al., 2017). Almost all teachers hope to help students form a GMCS in mathematics teaching. In order to achieve this goal, researchers and educators have made a long-term exploration and are continuously studying this issue. Based on previous studies, this study selected 82 Calculus concepts and 43 top students, analyzed their MCS with the social network method, based on a general perspective that the MCS of top students in mathematics must be good (Tsai, 2001; Wang and Wang, 2004; Wang, 2013).

The analysis that aGMCS should have the following features: (1) There are more connections between MK, especially those with the higher connection tightness. (2) There is more MK connected with others. (3) There is more MK directly linked to other MK. (4) There are a number of basic MK or MK with a high degree of inclusion connecting other MK and exerting an important impact. (5) Most of the MK interconnect with each other, especially the concepts with high connection tightness so that the number of subgroups is relatively small.

These findings although different, look similar to those previously revealed. (1) Although previous researchers mentioned that the connections between MK in a GMCS are extensive, they did not mention those connections with high tightness. There is no clear explanation of what these connections look like. (2) It is believed that abstract and inclusive knowledge is found in the center of a GMCS and are widely linked to other concepts. The number of centers are not specified. This study found there could be more than one MK center in a GMCS. (3) Even though the previous study mentioned the connection between the basic and inclusive MK and other MK, they did not indicate the type of connection. This study found that most were direct connections. As a result, concepts of a basic and the higher inclusive level have a greater impact in a GMCS. (4) Previous studies suggest that a GMCS is composed of multiple subsets, while we found that the subsets in a GMCS were actually very few. This suggests that most MK is closely and tightly linked in s GMCS.

In fact, we conducted a parallel study and obtained most of the above results in 2018 (Yang et al., 2018). A new study was undertaken as students selected for the previous study were high school students with a low mathematical comprehension. Even so, the high school students’ MCS similar to that of university students and analyzing the MCS of top university students in mathematics further might reveal more refined results.

Based on the characteristics achieved from a typical GMCS in this study, we believe that to help students form a GMCS, teachers should guide their students to establish a wide connection of MK especially direct connections, so that students form a tight and flexible MCS. At the same time, teachers should attach importance to all fundamental MK and knowledge with a higher inclusion, and help students establish an extensive connection between the fundamental MK and others, so as to form more MK accumulation points in their MCS.

The findings of this study enrich the existing research results, expand the research field of MCS, and point out the direction and goal of educators to help students form a GMCS. This study also revealed that it was feasible and effective to apply the social network analysis method to the study of MCS. Therefore, this study promotes the study of MCS, especially the study of the characteristics of knowledge organization in GMCS.

Objectively speaking, this study also had some shortcomings The subjects we chose were students from Chinese universities only. This would inevitably affect the accuracy of the results to some extent. Secondly, the materials we used included only some mathematical concepts and did not include other MK, such as mathematical formulas, theorems, and propositions. It was hard to avoid the suspicion of generalizing the mathematical concept as the object of study. It is therefore necessary to increase the number of studies from different regions and use more extensive research materials in the follow-up study, which will help to obtain more accurate and stable research results.

## Author Contributions

ZY designed the study and guided the investigation and the data analysis. MZ collected the data and did the calculations. YZ wrote the research report. KW translated Chinese into English.

## Conflict of Interest Statement

The authors declare that the research was conducted in the absence of any commercial or financial relationships that could be construed as a potential conflict of interest.
